# Combat Diabetic Nephropathy: From Pathogenesis to Treatment

**DOI:** 10.1155/2014/207140

**Published:** 2014-03-11

**Authors:** Tomohito Gohda, Akira Mima, Ju-Young Moon, Keizo Kanasaki

**Affiliations:** ^1^Division of Nephrology, Department of Internal Medicine, Juntendo University Faculty of Medicine, Tokyo 113-8421, Japan; ^2^Department of Nephrology, Graduate School of Medicine, Institute of Health Biosciences, University of Tokushima, Tokushima 770-8503, Japan; ^3^Division of Nephrology, Department of Internal Medicine, Kyung Hee University Hospital at Gangdong, College of Medicine, Kyung Hee University, Sangil-dong 149, Gangdong-gu, Seoul 134-727, Republic of Korea; ^4^Department of Diabetology & Endocrinology, Kanazawa Medical University, Uchinada, Ishikawa 920-0293, Japan

Diabetic nephropathy (DN) is the most common microvascular complication of diabetes and remains one of the leading causes of end-stage renal disease worldwide. The pathogenesis of DN was thought of as very simplistic up to several decades ago. Basically, the main risk factors contributing to the development and/or progression of DN are hyperglycemia, hypertension, dyslipidemia, and so on. In fact, Steno-2 study demonstrated that multifactorial therapies in patients with type 2 diabetes (T2D) successfully delayed the onset and retarded macrovascular and microvascular complications. However, the prevalence of nephropathy among patients with T2D has not fully decreased at this moment. Understanding other molecular mechanisms involved in DN is urgently needed for provision of better care for the patients.

In this special issue, original as well as review articles regarding the pathophysiologic mechanisms which are involved in the development and/or progression of DN were invited. Finally, we accepted six papers including three excellent review articles for the publication of this special issue after careful consideration.

In a research article of biomarker research, F.-q. Chen et al. (China Medical University, Shenyang, China) performed the multiple regression analysis and principle component analysis in patients with various stages of Chinese type 2 diabetes (112 normo-, 93 micro-, and 56 macroalbuminuric patients) to examine the predictive value of albuminuria as the dependent variable. They concluded principle component for hs-CRP, serum TNF*α*, serum amyloid-A (SAA) and urinary MCP-1 was determinant for prediction of albuminuria in addition to HbA1c.

In another biomarker research, N. A. Seman et al. (Karolinska University Hospital, Stockholm, Sweden) measured the plasma pentraxin 3 (PTX3) levels in Malay type 2 diabetic patients with (*n* = 102) and without nephropathy (*n* = 91). They clearly demonstrated the levels of PTX3 differ according to the presence or absence of nephropathy, obesity, and sex.

C. Gao et al. (affiliated Hospital of Luzhou Medical College, Sichuan, China) have tested the efficacy of MG132, a ubiquitin protease inhibitor in the treatment of DN using experimental type 1 diabetes. They showed that this drug might alleviate the kidney damage by inhibiting Smad7 ubiquitin degradation and TGF-*β* activation.

Finally, three review articles are especially exceptional. K. Yamahara et al. (Shiga University of Medical science, Shiga, Japan) proposed that autophagy insufficiency has some potential role in the progression of DN although it remains unclear whether autophagy activation is beneficial in all stages of DN. T. Nakagawa et al. (Kyoto University Graduate School of Medicine, Kyoto, Japan) proposed the importance of appropriate VEGF for normal renal physiology especially in diabetic condition. They emphasized that the coupling of VEGF-NO axis (both appropriate VEGF and NO levels) is required to maintain normal angiogenesis. S. Hagiwara et al. (Baker IDI Heart and Diabetes Institute, Melbourne, Australia) wrote a nice review of the literature about the role of microRNA in DN, especially focused on renin-angiotensin system, AGE/RAGE signaling, and oxidative stress. Exciting data demonstrating the potential to attenuate or even reverse renal disease in experimental diabetes by restoring the expression of a dysregulated miRNA provide impetus for further studies in this area.

In summary, we still have a long way to go before resolution of combat DN but believe this special issue provides some clues to combat DN in the clinical setting. We would be most grateful if this special issue has been helpful for not only nephrologists and endocrinologists but also all internal physicians.

*Tomohito Gohda* *Tomohito Gohda* *Akira Mima* *Akira Mima* *Ju-Young Moon* *Ju-Young Moon* *Keizo Kanasaki* *Keizo Kanasaki*

